# Water Stress Enhances the Progression of Branch Dieback and Almond Decline under Field Conditions

**DOI:** 10.3390/plants9091213

**Published:** 2020-09-16

**Authors:** Carlos Agustí-Brisach, David Moldero, María del Carmen Raya, Ignacio J. Lorite, Francisco Orgaz, Antonio Trapero

**Affiliations:** 1 Departamento de Agronomía, ETSIAM, Universidad de Córdoba, Campus de Rabanales, Edif. C4, 14071 Córdoba, Spain; ag2raorm@uco.es (M.d.C.R.); trapero@uco.es (A.T.); 2 Instituto de Agricultura Sostenible (IAS), Consejo Superior de Investigaciones Científicas (CSIC), Campus Alameda del Obispo, Avda Menéndez Pidal s/n, 14004 Córdoba, Spain; dmoldero@ias.csic.es (D.M.); orgaz@ias.csic.es (F.O.); 3 IFAPA-Centro Alameda del Obispo, Junta de Andalucía, Avda Menéndez Pidal s/n, 14004 Córdoba, Spain; ignacioj.lorite@juntadeandalucia.es

**Keywords:** fungal trunk pathogens, occurrence, *Prunus dulcis*, water deficiency, weakened hosts

## Abstract

Branch dieback and tree decline have been described as a common complex disease worldwide in woody crops, with Botryosphaeriaceae and Diaporthaceae being considered the most frequent fungi associated with the disease symptoms. Their behaviour is still uncertain, since they are considered endophytes becoming pathogenic in weakened hosts when stress conditions, such as water deficiency occur. Therefore, the main goal of this study was to determine if water stress enhances general decline on weakened almond trees subjected to different irrigation treatments under natural field conditions. In parallel, the occurrence of fungal species associated with almond decline was also determined in relation to disease progression by fungal isolation, and morphological and molecular based-methods. The symptoms of branch dieback and general decline were observed over time, mainly in the experimental plots subjected to high water deficiency. Botryosphaeriaceae were the most consistently isolated fungi, and *Botryosphaeria dothidea* was the most frequent. *Collophorina hispanica* was the second most frequent species and *Diaporthe* and *Cytospora* species were isolated in a low frequency. Most of them were recovered from both asymptomatic and symptomatic trees, with their consistency of isolation increasing with the disease severity. This work reveals the need to elucidate the role of biotic and abiotic factors which increase the rate of infection of fungal trunk pathogens, in order to generate important knowledge on their life cycle.

## 1. Introduction

Almond [*Prunus dulcis* (Mill.) D.A. Webb] represents the second highest woody crop by acreage in Spain after cultivated olive (*Olea europaea* subsp. *europaea* L.). To date, Spain currently leads the world in almond cultivation, with 657,768 hectares of cultivated almond (34% of the global surface) and 339.033 tonnes of production [1,2]. In this country, Andalusia region (southern Iberian Peninsula) represents the 30% of the Spanish cultivated almond surface [2].

Until recently, almond crop has been associated with traditional dry farming systems in marginal areas of southern Spain with unfavorable conditions to produce high yields. However, due to the global economic impact of almond kernels, as well as the necessity to find extensive alternative crops in Andalusia, almond plantings are increasingly being established in regions with better favorable conditions (i.e., moderate-warm temperatures, high humidity, irrigation-water resources, etc.). This change also involves different cultural practices than those used in traditional systems which are more likely to increase yields. They include dense planting, high levels of irrigation-water and fertilization, pruning- and harvest-mechanization, and a high number of pesticide treatments preventing pest and plant diseases [3]. As a consequence of this new scenario, the occurrence of secondary almond diseases, as well as emerging ones, have been reported recently in the new almond growing regions across the Guadalquivir Valley in Andalusia region [3,4,5].

Among the emerging diseases already described in this geographic area [3,4,5], a new tree decline syndrome stands out in the new intensive almond plantings. It includes a broad diversity of symptoms, such as gummosis, shoot blight, defoliation, branch dieback, canker formation, internal wood discoloration and tree death. The first studies determining its etiology suggest that it is a complex disease probably associated with Botryosphaeriaceae Theiss. and Syd. fungi, among other secondary pathogens [5]. On the other hand, other syndromes, such as a branch dieback and cankers associated with *Diaporthe amygdali* (Delacr.) Udayanga, Crous and K.D. Hyde, or the foamy canker, have also been observed in the new almond plantings in Andalusia. In particular, foamy canker always occurs when the vigorous hybrid Garnem is used as rootstock [5], but the causal agent of these syndrome has not yet been described anywhere, due to the impossibility of reproducing the symptoms with the microorganisms isolated from the diseased trees [5,6]. However, the etiology of all these syndromes in the environmental conditions of Andalusia is still uncertain, since little attention has been given to their low occurrence, until recently. The only previous studies describing symptoms of almond decline in Spain were conducted in Mallorca (Balearic Islands, western Mediterranean Sea) [7,8,9]. These authors indicated that fungi belonging to Botryosphaeriaceae were the main causal agents associated with the disease, among other secondary fungal species belonging to the genera *Collophorina* (=*Collophora*) Damm and Crous, *Diaporthe* Nitschke, *Eutypa* Tul. and C. Tul. or *Phaeoacremonium* W. Gams, Crous and M.J. Wingf. Branch dieback and tree decline has been described as a common syndrome worldwide in a broad diversity of woody crops including grapevine [10,11,12], olive [13,14,15] and tree nuts [6,15,16,17]. In any cases, the main fungal species associated with tree decline belongs to Botryosphaeriaceae and/or Diaporthaceae Höhn. ex Wehm., with the first ones being the most aggressive [6,9,15,16,17,18]. However, the role of this wide diversity of fungi that has been causing tree decline is still uncertain. Most of the fungi are characterized by remaining latent in the infected tissues for a long period of time (endophytic phase), but they become pathogenic in weakened hosts when stress conditions occur [19,20]. In fact, it is rare to find weakened or stressed trees that are not infected by dieback and canker fungi, while their occurrence and aggressiveness is low in healthy plants [19].

In this sense, previous studies evaluating the effect of water stress on development of canker diseases have been conducted under semi-controlled conditions. Crist and Schoeneweiss [21] demonstrated that canker formation and colonization of bark and wood on birch tree (*Betula alba* L.) occurred when seedlings inoculated with *Botryosphaeria dothidea* (Moug.) Ces. and De Not. were subjected to defoliation stress, increasing in severity with time of exposure to stress. Later studies with this same pathogen also demonstrated that the lesions developed on inoculated plants of peach [*Prunus persica* (L.) Batsch] were larger on water-stressed plants in comparison with those on non-stressed ones [22]. Similar studies have also been conducted to determine the effect of water stress on the aggressiveness of other pathogens, which are different to those described previously. For example, Maxwell et al. [23] evaluated the influence of water stress on Septoria canker, caused by *Septoria musiva* Peck in Populus stems. This study showed that cankers on inoculated water-stressed trees were significantly larger than those on non-stressed ones.

However, to date, there is no scientific evidence on the question of whether water stress could enhance the progression of branch dieback and general decline on weakened almond trees under field conditions. Since the occurrence of decline syndromes is growing in the new almond plantings in southern Spain along the last few years [5], determining whether water stress enhances the incidence and severity of almond decline is essential. The current scenario that we face to in the new almond plantings is subjected to two-limiting conditions, which could favor the disease development, including; (1) the typical environmental conditions in southern Spain are characterized by scarce rains and warm temperatures during summer (from May to September), which predispose plants to water deficiency for a long time; and (2) the need to optimize water-irrigation treatments within the frame of eco-friendly agriculture towards a sustainable use of water resources. Therefore, the main goal of this study was to determine whether water stress enhances the general decline of weakened almond trees, subjected to different irrigation treatments, under natural field conditions. In parallel, the occurrence of fungal species, associated with branch dieback and almond decline, was also determined in relation to the disease progress by fungal isolation, and morphological and molecular based-methods for their identification.

## 2. Results

### 2.1. Effect of Water Stress on Branch Dieback of Almond under Natural Field Conditions

**Figure 1:**
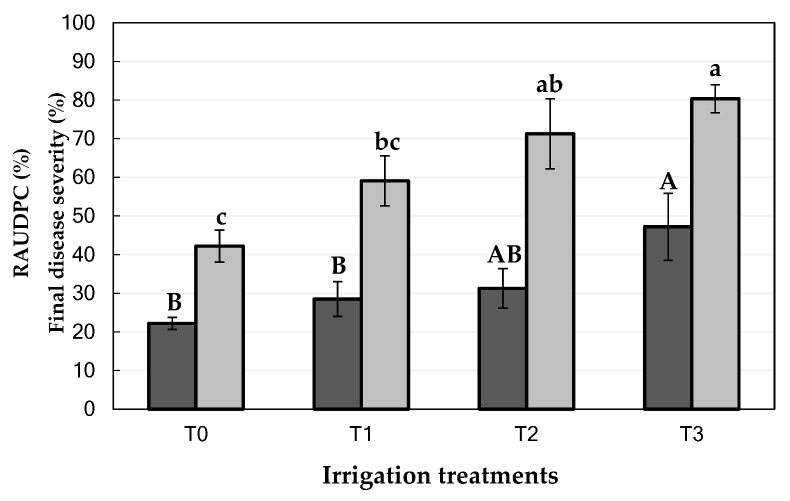
Disease severity [RAUDPC (%; dark grey columns) and Final disease severity (%; light grey columns)] of branch dieback of almond trees under natural conditions in an experimental field (Córdoba, Andalusia region, southern Spain) subjected to four irrigation treatments from April 2013 to October 2019 (T0: Control; T1: Moderate Regulated Deficit Irrigation; T2: Moderate Sustained Deficit Irrigation; T3: Severe Regulated Deficit Irrigation). The disease severity assessments were conducted from June 2018 (next spring after first symptoms of branch dieback occur) to September 2019 (end of the experiment). For each disease parameter, columns represent the means of sixteen trees and vertical bars are the standard errors of the means. Columns with different capital or lowercase letters differ significantly for RAUDPC, or Final disease severity, respectively, according to Fisher’s LSD test at P = 0.05.

**Figure 2:**
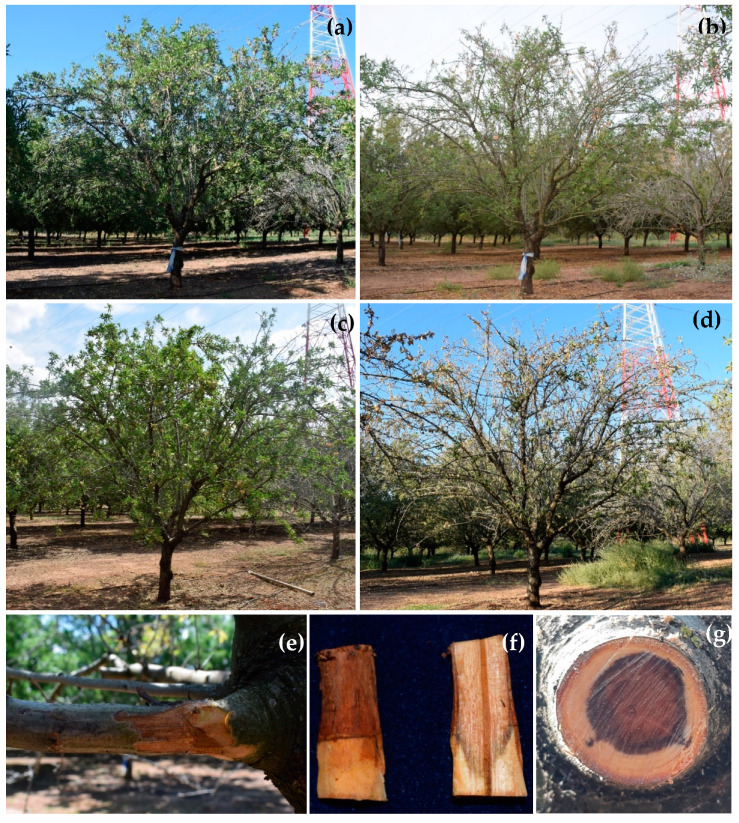
Disease progress and symptoms of branch dieback on one almond tree subjected to Severe Regulated Deficit Irrigation (T3) monitored from June 2018 to September 2019. Assessment times and rating-scales values were: (**a**) June 2018-2.0; (**b**) September 2018-3.0; (**c**) June 2019-3.0; (**d**) September 2019-4.0; (**e**–**g**) symptoms of branch dieback and internal wood discoloration in affected branches.

**Figure 3:**
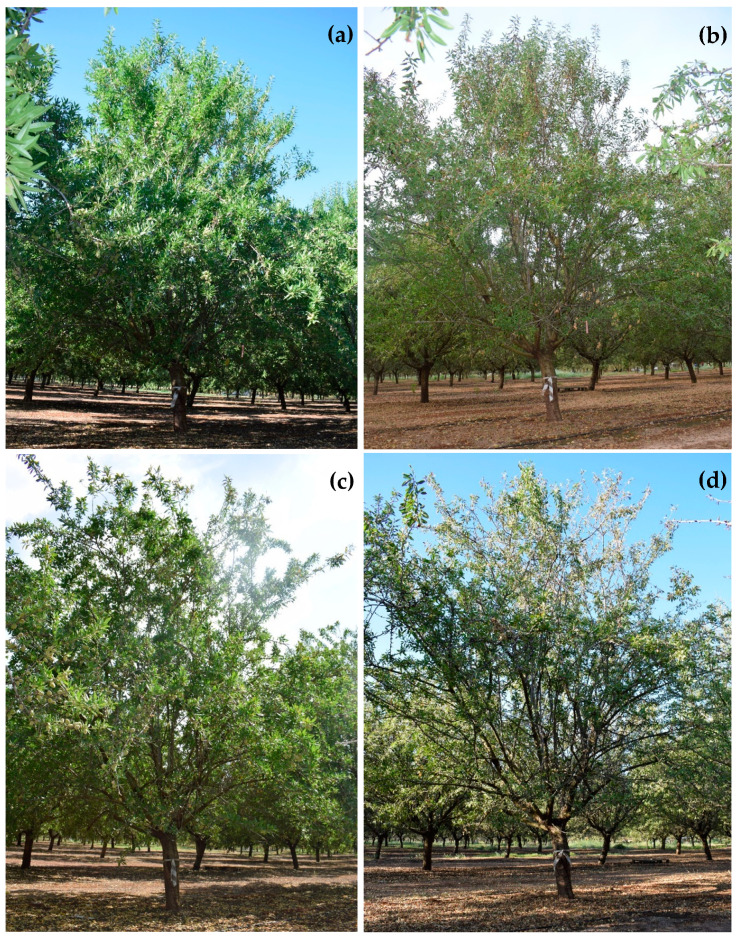
Disease progress on one almond tree from Control (T0) monitored from June 2018 to September 2019. Assessments times and rating-scales values were: (**a**) June 2018-0.0; (**b**) September 2018-1.0; (**c**) June 2019-1.0; (**d**) September 2019-2.0.

At the beginning of the evaluation period (June 2018), the number of almond trees of each category among the 80 evaluated trees was as follow: Category 0 = 34 trees 0 (asymptomatic trees with 0% of affected surface by branch dieback), Category 1 = 30 trees (<25% of affected surface by branch dieback), Category 2 = 8 trees (25–50% of affected surface by branch dieback); Category 3 = 1 tree (51–75% of affected surface by branch dieback); Category 4 = 0 trees (76–90% of affected surface by branch dieback); and Category 5 = 7 trees (>90% affected surface by branch dieback or dead trees). In general, Disease Severity (DS) progresses in significantly higher values of relative area under the disease progress curve (RAUDPC; *P* = 0.0173) and final disease severity (*P* = 0.0012) when almond trees were subjected to T3 (Severe Regulated Deficit Irrigation; RAUDPC = 47.2 ± 8.7%; Final disease severity = 80.4 ± 3.6%), followed by T2 (Moderate Sustained Deficit Irrigation; RAUDPC = 31.3 ± 5.1%; Final disease severity = 71.3 ± 9.1%) and T1 (Moderate Regulated Deficit Irrigation; RAUDPC = 28.5 ± 4.5%; Final disease severity = 59.1 ± 6.5%) (Figure 1 and Figure 2). Almond trees used as control (T0) showed the lowest values of RAUDPC (22.2 ± 1.5%), as well as the lowest values of final disease severity (42.2 ± 4.1%) (Figure 1 and Figure 3). Control trees did not show internal wood discoloration.

### 2.2. Occurrence, Consistency and Frequency of Isolated Fungi

**Figure 4:**
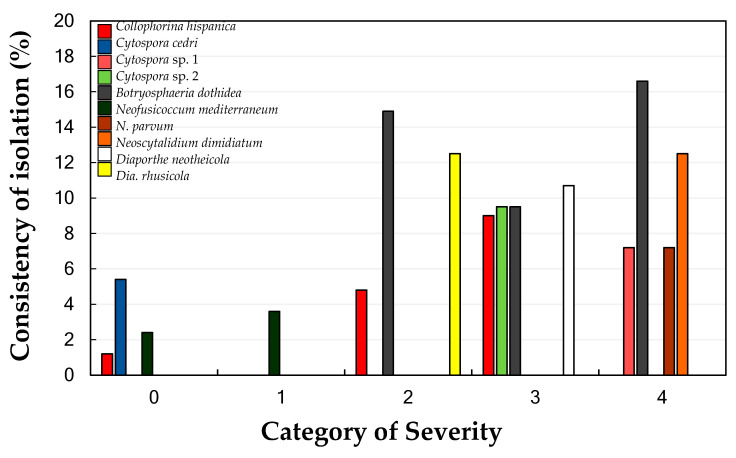
Consistency of isolation (Y-axis; Av. %) of the fungal species identified in this study associated with branch dieback of almond in each category of severity (0 = 0%, 1 = < 25%, 2 = 25–50%, 3 = 51–75%, 4 = 75–90% of affected surface by branch dieback) in the whole of the experiment. For each category and fungal species, columns represent the total consistency of isolation along the two years (2018–2019), in which the disease severity was evaluated [Consistency of isolation = (Nº. of positive wood pieces/168) × 100; where 168 is the total attempts of isolation (wood pieces) per category of disease severity in the whole of the experiment obtained as follow: 21 wood pieces per tree × 2 trees of each disease category × 2 years of evaluation × 2 sampling times per year].

Fungal species, associated with branch dieback and almond decline, were isolated from all the categories of severity evaluated, with the exception from the trees belonging to the category 5 from which only saprophytes (i.e., *Alternaria* spp. Nees, *Penicillium* spp. Link, *Sordaria* spp. Ces. and De Not., etc.) were recovered. The consistency of isolation of each isolated fungi is shown in Table 1. In general, the occurrence of fungal species and the consistency of their isolation increased with the DS, with trees belonging to the categories 3 and 4 showing the highest number of fungal species, as well as the highest consistency of isolation. However, three fungal species were isolated from trees belonging to category 0, while only one fungal species was recovered from trees belonging to category 1. The consistency of isolation in these two categories was somewhat lower (≤5.4%) than those obtained from the remaining ones (up to 16.6%) (Figure 4). The total fungal biomass in the sampled trees per category was three fungal species in the trees belonging to Category 0, one fungal species in the trees belonging to Category 1, three fungal species in the trees belonging to the Category 2, and four fungal species in the trees belonging to the Category 3 and 4. Therefore, there was no linear correlation between the in planta abundance (biomass) of the studied fungal species and the severity of the category of severity (*r* = −0.6455; *P* = 0.2394).

Fungal species, belonging to Botryosphaeriaceae, were the most frequent and they also showed the highest consistency of isolation of the whole of the experiment (Table 1). *Botryosphaeria dothidea* was the most frequent species, since it was isolated from the 50.0% of the sampled trees, from trees belonging to the categories 2, 3 and 4. This was followed by *Collophorina hispanica* (Gramaje, Armengol and Damm) Damm and Crous, which was isolated from the 41.6% of the sampled trees, from trees belonging to the categories 0, 2 and 3. The species belonging to *Cytospora* Ehrenb. were also isolated from trees belonging to the categories 0 (*Cytospora cedri* Syd., P. Syd. & E.J. Butler), 3 (*Cytospora* sp. 2) and 4 (*Cytospora* sp. 1) with a low frequency (8.3%). The frequency of the remaining species was also low [*Dia. neotheicola* A.J.L. Phillips and J.M. Santos (8.3%), *Dia. rhusicola* Crous (8.3%), *N. mediterraneum* Crous, M.J. Wingf. and A.J.L. Phillips (16.7%), *N. parvum* (Pennycook and Samuels) Crous, Slippers & A.J.L. Phillips (8.3%) and *Neoscytalidium dimidiatum* (Penz.) Crous and Slippers (8.3%)]. Co-infections in the same tree and sampling moment occurred only one time for the following combinations: *C. hispanica* and *Cytospora* sp.; and *Cytospora* sp. and *N. parvum*. 

### 2.3. Molecular Identification of Isolated Fungi

For all Datasets, the topology obtained by Maximum Parsimony (MP) was confirmed with those obtained by BI analysis. The model used in BI analysis, and the gene boundaries, the number of total characters (T), parsimony-informative characters (PI), parsimony-uninformative characters (PNI) and conserved sites (C) processed in each maximum parsimony analysis, as well as TL, consistency index (CI), retention index (RI), homoplasy index (HI) and rescaled consistency index (RC) values obtained from the one most parsimonies trees in each Dataset are shown in Table 2.

**Figure 5:**
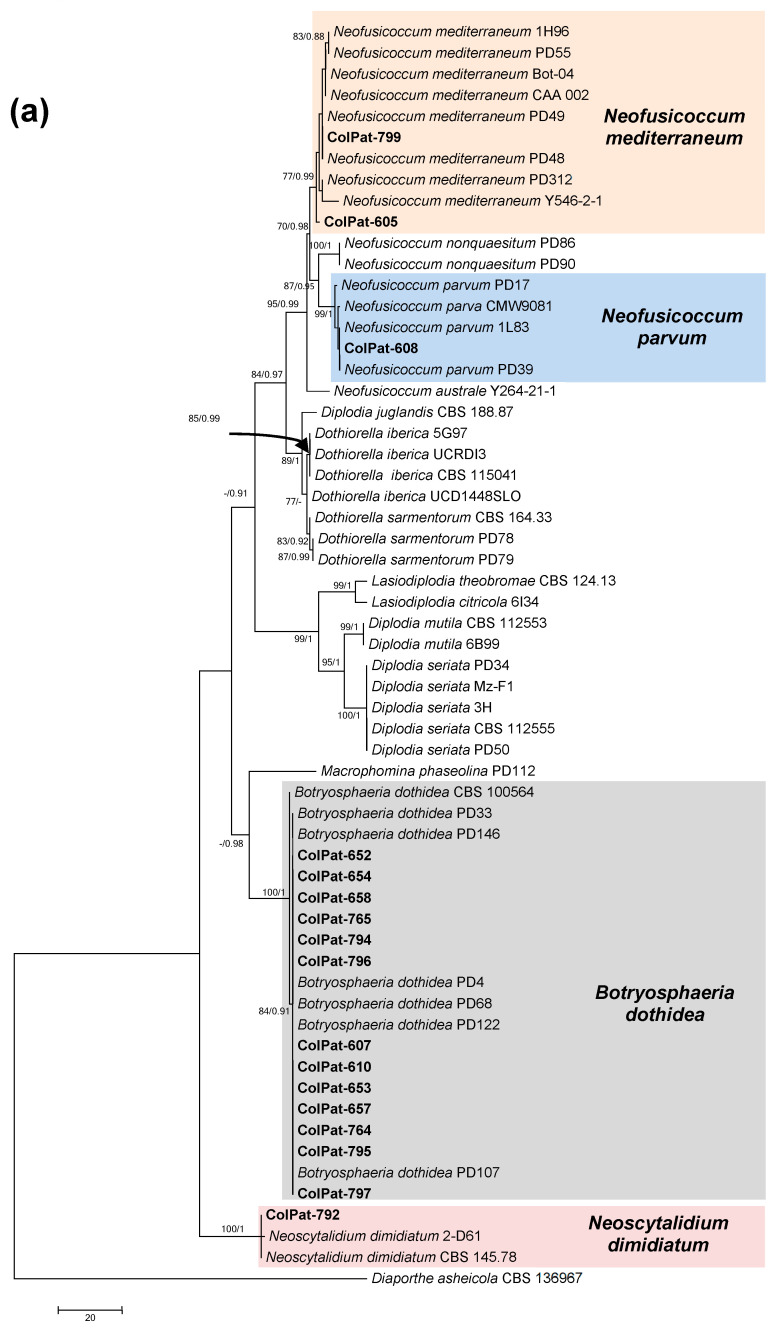

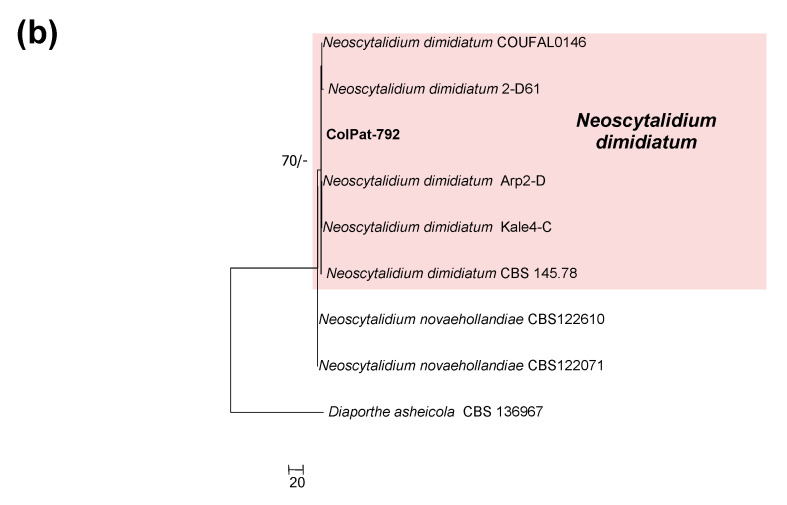
(**a**) The first of the four most parsimonious trees (TL = 375; CI = 0.677; RI = 0.940; HI = 0.323; RC = 0.636) obtained by Maximum Parsimony (MP) analyses of combined ITS+TUB sequence alignment of species belonging to Botryosphaeriaceae; (**b**) One of the nine MP trees (TL = 258; CI = 0.854; RI = 0.833; HI = 0.143; RC = 0.714) obtained using the combined ITS+TUB+EF sequence alignment of species belonging to *Neoscytalidium*. Bootstrap support values [MP, >70%] and Bayesian posterior probabilities [PP, >0.8] are shown at the nodes. *Diaporthe asheicola* L. Lombard & Crous CBS 136967 was used as the outgroup. Studied isolates in bolt.

Botryosphaeriaceae analyses (*Dataset I-A*). Most of the Botryosphaeriaceae isolates (13 out of 17 isolates) were grouped in a well-supported clade with GenBank reference sequences of *B. dothidea* [bootstrap support (BS; %)/Bayesian posterior probability (PP):100/1.00]. The remaining isolates were identified as *N. mediterraneum* (ColPat-605 and ColPat-799; BS/PP:77–83/0.99–0.88), *N. parvum* (ColPat-608; BS/PP:99/1.00), and *Neoscytalidium dimidiatum* (Penz.) Crous and Slippers (ColPat-792) (BS/PP:100/1.00) (Figure 5a). To confirm the identification of this last isolate, an additional phylogeny was conducted by means the combined alignment of ITS and EF loci, including reference isolates of *Neoscytalidium novaehollandiae* Pavlic, T.I. Burgess and M.J. Wingf. (*Dataset I-B*). The MP analyses showed nine most parsimonious, and one of those is shown in Figure 5b. 

**Figure 6:**
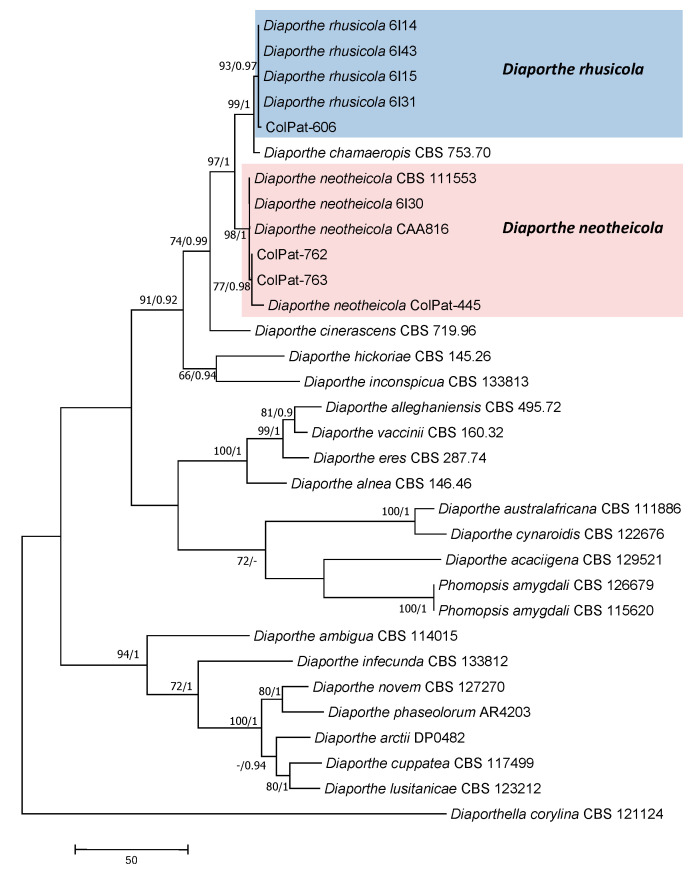
The most parsimonious tree (TL = 1046; CI = 0.551; RI = 0.769; HI = 0.449; RC = 0.424) obtained by Maximum Parsimony analyses of the combined EF+TUB+ITS sequence alignment of species belonging to Diaporthaceae. Bootstrap support values [MP, >70%] and Bayesian posterior probabilities [PP, >0.8] are shown at the nodes. *Diaporthella corylina* Lar.N. Vassiljeva CBS 121124 was used as the outgroup. Studied isolates in bolt.

Diaporthaceae analyses (*Dataset II*). Our isolates clustered in two well-supported clades with reference sequences of *Dia. neotheicola* (ColPat-762 and ColPat-763; BS/PP:98/1.00) and *Dia. rhusicola* (ColPat-606; BS/PP:99/1.00) (Figure 6).

**Figure 7:**
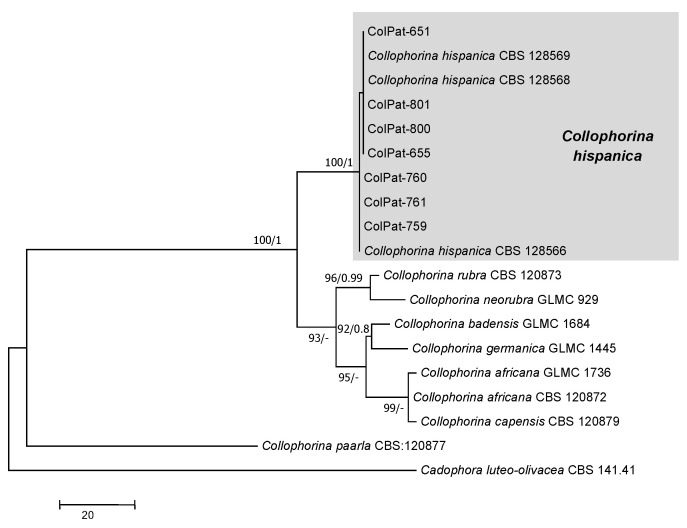
The first of the 10 most parsimonious tree (TL = 334; CI = 0.850; RI = 0.919; HI = 0.150; RC = 0.782) obtained by Maximum Parsimony analyses of the combined ITS + EF + GADPH sequence alignment of species belonging to Tympanidaceae. Bootstrap support values [MP, >70%] and Bayesian posterior probabilities [PP, >0.8] are shown at the nodes. *Cadophora luteo-olivacea* (J.F.H. Beyma) T.C. Harr. and McNew CBS 141.41 was used as the outgroup. Studied isolates in bolt.

Tympanidaceae analysis (*Dataset III*). All the isolates belonging to Tympanidaceae clustered together in a well-supported clade with GenBank reference sequence of *Collophorina hispanica* (=*Collophora hispanica*; BS/PP:100/1.00) (Figure 7).

**Figure 8:**
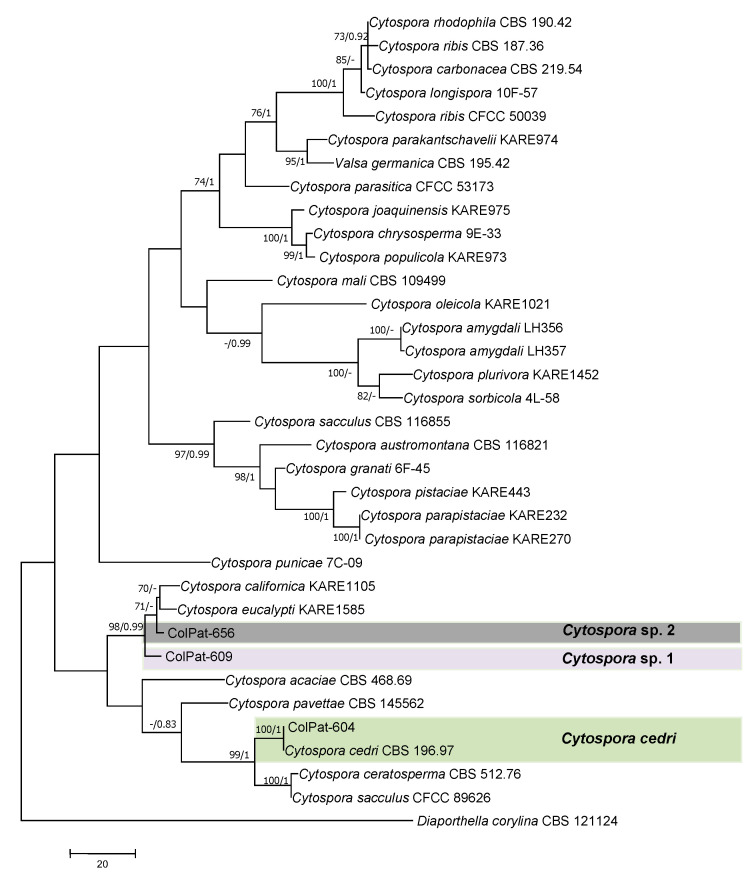
The most parsimonious tree (TL = 743; CI = 0.469; RI = 0.742; HI = 0.531; RC = 0.348) obtained by Maximum Parsimony analyses of the combined ITS+EF sequence alignment of species belonging to Valsaceae. Bootstrap support values [MP, >70%] and Bayesian posterior probabilities [PP, >0.8] are shown at the nodes. *Diaporthella corylina* CBS 121124 was used as the outgroup. Studied isolates in bolt.

Valsaceae analysis (*Dataset IV*). Among the three isolates belonging to Valsaceae included in this study, only one (ColPat-604) was grouped in a well-supported clade with a GenBank reference sequence of *Cytospora cedri* Syd., P. Syd. and E.J. Butler (BS/PP:100/1.00). However, it was not possible to distinguish the remaining two isolates (ColPat-609 and ColPat-656) at the species level into the genus *Cytospora*, and they were identified as *Cytospora* sp. 1 (ColPat-609) and *Cytospora* sp. 2 (ColPat-656) (Figure 8).

## 3. Discussion

Studying whether the effect of abiotic factors, such as water stress enhances the incidence and development of branch dieback and decline syndromes on weakened trees, is essential in improving our understanding of the endophytic behaviour of fungi associated with this complex disease. In fact, to date, the role of the fungal trunk pathogens, causing tree decline, is still uncertain, since their aggressiveness could vary markedly depending on abiotic (i.e., ecological, environmental and agronomical aspects) and/or biotic (i.e., plant-pathogen interactions) factors. Consequently, several authors consider that most of the fungal trunk pathogens are secondary or opportunistic, causing damage when biotic or abiotic circumstances occur [11,19,24,25]. 

The environmental and agronomic conditions regarding the availability of irrigation-water resources in southern Spain could be a limiting factor, enhancing the development of branch dieback and decline syndromes on fruit and nut crops. Therefore, we have evaluated the effect of water stress enhancing the disease development on weakened almond trees under natural field conditions. The first symptoms of branch dieback occurred in late-summer autumn 2017 in an eight-year old experimental field, subjected to four different irrigation treatments, since 2013. The symptoms included branch dieback, canker formation, internal wood discoloration and general decline (Figure 2d–g) were observed mainly in the experimental plots subjected to high water-stressed conditions (T2, T3). In fact, the DS progress was significantly higher in almond trees subjected to T3 than in those subjected to T0 after two consecutive years of periodic evaluations. Our results are in accordance with those previously obtained by several authors under the control conditions, which showed that stem cankers, developed by *B. dothidea* or *S. musiva* on water-stressed plants of peach, or *Populus*, respectively, were higher than those developed on non-water-stressed plants [22,23]. On the other hand, almond trees under full irrigation (T0) also showed minimum levels of dieback symptoms. Although, no internal wood discoloration was observed, several fungal species, such as *C. hispanica*, *Cy. cedri* and *N. mediterraneum* were isolated from those trees. These results reinforce the hypothesis that these fungi could cause latent infections in asymptomatic or lesser-symptomatic trees. In parallel, the conclusions obtained in this study should be considered to discard the high levels of irrigation water as potential abiotic factor associated with the prevalence of the disease in the newly established almond growing regions in southern Spain, as we initially hypothesized in the introduction. To the best of our knowledge, this is the first approach, which has demonstrated the endophytic behaviour of fungal trunk pathogens on weakened trees, subjected to water stress under natural field conditions. 

Concerning the occurrence of fungal species associated with branch dieback and almond decline, the following seven species belonging to four different families were identified: Botryosphaeriaceae: *B. dothidea*, *N. mediterraneum*, *N. parvum* and *Neoscytalidum* sp.; Diaporthaceae: *Dia. neotheicola* and *Dia. rhusicola*; Tympanidaceae: *C. hispanica*; and, Valsaceae: *Cytospora cedri* and *Cytospora* spp. Among them, *B. dothidea*, *C. hispanica*, *Dia*. *neotheicola*, *N. mediterraneum* and *N. parvum* have been previously described associated with branch dieback and decline on weakened almond trees in Spain [7,8,26]. Moreover, the pathogenicity of most of these species has been previously demonstrated in almond trees in Spain [5,8,9,26]. On the other hand, *Dia. rhusicola* and species belonging to *Cytospora* and *Neoscytalidium* genera, are associated with branch dieback and tree decline in other nut crops, such as English walnut or pistachio [6,15,16,17], but to our knowledge, these species have not been previously reported in association with branch dieback and almond decline in Spain. However, their pathogenicity to almond should be demonstrated in the future to confirm they are canker pathogens of almond.

Botryosphaeriaceae were the most frequent isolated fungi and they also showed the highest consistency of isolation in the whole of the experiment, with *B. dothidea* being the species most frequently isolated. The differences in consistency of isolation of Botryosphaeriaceae fungi from weakened almonds can occur, depending on the scenario where the surveys are conducted, but in general, *B. dothidea* and *Neofusicoccum* species are usually the most frequent [9,25]. Likewise, according to the literature, our results also suggest that Botryosphaeriaceae spp. found on weakened almond trees are able to endanger the productivity and longevity of orchards in Spain, as well as in other countries [9].

Among Botryosphaeriaceae fungi, notice that *B. dothidea* has been reported worldwide causing canker diseases in a broad range of woody crops, including different *Prunus* spp. [6,7,27]. However, the role of this fungus as a trunk pathogen is still uncertain, given it has been reported as a latent pathogen of global importance for its endophytic behaviour in woody plant health [20]. In fact, studies conducted recently in southern Spain, which compared the pathogenicity of *B. dothidea* on inoculated detached and attached shoots of almond, the English walnut and pistachio, demonstrated that, in every case, the fungus is significantly higher aggressive on detached shoots than on attached [5,16,17]. It was confirmed that *B. dothidea* could remain latent in woody plants until trees become weakened as a consequence of different biotic and/or abiotic factors. 

With respect to Diaporthaceae, *Dia. neotheicola* and *Dia. rhusicola* showed a low consistency of isolation and their occurrence was also low. These two species have been previously reported, associated with branch dieback and shoot blight of English walnut in California [28] and southern Spain [16], and the first one was also isolated from pistachio in southern Spain [17] and recently reported associated with twig cankers and shoot blight of almond in Spain [26]. Usually, Diaporthaceae species occur simultaneously with Botryosphaeriaceae in the same orchards, with Botryosphaeriaceae being always the most frequent [16,17,28]. In addition, studies conducted in California by Agustí-Brisach et al. [29] suggest that coinfections between Botryosphaeriaceae and Diaporthaceae species result in antagonistic interactions on infection and disease development on English walnut. But, *Dia. amygdali*, which is a common species associated with branch dieback and cankers of almond [5,7], was not found in this experiment. 

It is interesting to note that, in this study, *C. hispanica* was the second most frequent species isolated from weakened almond trees after *B. dothidea.* Our results are in concordance with those found by Olmo et al. [8], who indicated that this slow-growing species is common in declined almond trees. However, it is usually excluded in the diagnosis process probably because its presence goes unnoticed, due to its slow growth [8]. 

Finally, *Cytospora* species were also isolated in low consistency from the trees of three different categories of severity. According to our results, *Cytospora* spp. have also been reported associated with canker diseases in weakened tree nuts (English walnut and pistachio) in southern Spain showing less frequency and aggressiveness, and often simultaneously with Botryosphaeriaceae and/or Diaporthaceae fungi [16,17]. However, several *Cytospora* spp. have been already reported in California as canker pathogens of several fruit and nut crops including *Prunus* spp. such as almond, apricot or peach [30].

The isolations made during this study suggest that most of these fungi can occur in both asymptomatic and symptomatic trees, but their frequency of isolation increases with increasing DS. However, the consistency of isolation was low for all the species in the whole of the experiment, showing the highest values in weakened almond trees, belonging to categories 3 and 4 (51 to 90% of final disease severity). This information reinforces the hypothesis that the fungal species associated with tree decline could have a major endophytic behaviour, and its aggressiveness is probably enhanced by abiotic factors, such as water stress on previously infected and weakened trees. In general, studies on the etiology of fungal trunk diseases describe a broad list of fungi associated with the disease, but do not usually considering the strict pathogenic behaviour of each. Nevertheless, this work reveals the need to go on elucidating the role of biotic and abiotic factors, enhancing the infection of fungal trunk pathogens and disease development on woody crops towards generating important knowledge on their life cycle. Therefore, focus the research on such relevant challenge will provide a better understanding of the biology of fungi associated with tree decline syndrome. It will build a strong foundation for developing effective management approaches against the disease, by taking into consideration the optimum water management.

## 4. Materials and Methods 

### 4.1. Experimental Field, Irrigation Treatments and Experimental Design

The present study was conducted in a nine- to 10-years-old experimental field of almond cv. Guara grafted onto GF-677 rootstock (5.5 ha; 7 × 6 tree spacing; 238 trees/ha) belonging to the Andalusian Institute of Agricultural and Fisheries Research and Training (IFAPA in Spanish) Centre ‘Alameda del Obispo’ located in Córdoba (Andalusia region, Spain; 37.8ºN, 4.8ºW), whose soil was classified as a Typic Xerofluvent of sandy loam texture and exceeds 1.5 m depth. The climate of this region is the typical Mediterranean climate, characterized by hot and dry summers (Tª Av. 27.0 °C; Tª min Av. = 19.3 °C; Tª max Av. = 36.7 °C), mild winters, with 600 mm of annual rainfall average, concentrated from October to April (Tª Av. 13.0 °C; Tª min Av. = 7.9 °C; Tª max Av. = 21.1 °C). 

The experimental field used in this study was established in February 2009. Pruning for tree formation was done along the two first years, and then there never were pruning interventions. Control management strategies to prevent pest (Acetamiprid 20%; Deltametrine 2.5%) and diseases (Boscalid 26.7% + Pyraclostrobin 6.7%; Thiram 50%; Tebuconazole 50% + Tryfloxistrobin 25%) were done according to a treatment-calendar based on the weather conditions which could favour the typical almond pest and diseases of this area. Weeds were controlled by mowing and herbicide applications (Glyphosate 36%; Oxifluorphen 24%). Mineral fertilization was calculated and applied following the recommendations of the California Fertilization Guidelines for Almonds (https://apps1.cdfa.ca.gov/FertilizerResearch/docs/Almonds.html). Therefore, this experimental field is representative of the edapho-climatic characteristics and the standard crop management of the new almond plantings in Andalusia [31].

The irrigation system was formed by two pressure compensating drip irrigation laterals, spaced 1 m from the tree rows, and all the trees were fully irrigated until the irrigation treatments began, as described below. From April 2013 to October 2019 (before and along this present study), the experimental field was subjected to four irrigation treatments: (*i*) *Control* (T0): The trees were irrigated to cover their full water requirements (ET), which was calculated using the relationship between ground cover (GC) and a transpiration coefficient, proposed by Espadafor et al. [32]. An additional 15% of that quantity was supplied to account for the evaporation from emitter wet surfaces under the trees using Bonachela et al. [33] model; (*ii*) *Moderate Regulated Deficit Irrigation* (T1): seasonal irrigation was 65% of T0, but the deficit was mainly concentrated during kernel filling stage, where almonds are less affected by water stress. Specifically, the irrigation supplied was: 70% of T0 in spring; 40% in kernel filling stage (from middle-July to harvest period in middle-August) and 100% in the postharvest period; (*iii*) *Moderate Sustained Deficit Irrigation* (T2): This treatment consisted of 65% of T0 steadily throughout the irrigation season. In total, a similar amount of irrigation water to that of the T1 was supplied; and (*iv*) *Severe Regulated Deficit Irrigation* (T3): This treatment received 30% of seasonal irrigation in relation to T0, following a similar water allocation strategy as in T1. Irrigation was 40% of T0 in spring and after harvest, and only 15% during the kernel-filling stage.

A randomized complete block design, with four replicated blocks, each consisting of four irrigation treatments, was used in this experiment. There were 16 trees per treatment plot, from which the four central trees were used for experimental measurements and the remaining 12 ones served as guard line. Therefore, the experiment included a total of 256 trees (4 blocks × 4 irrigation treatments × 16 trees per elementary plot), from which 80 trees were evaluated. Weather data were collected from an automated weather station located at 300 m apart from the orchard.

### 4.2. Disease Severity Assessment and Data Analysis

In the summer-autumn of 2017, the experimental trees subjected to the different irrigation treatments started to weaken, showing the first symptoms of branch dieback and general decline. Since then, the progress of this syndrome was monitored over time by periodic assessments from June 2018 to September 2019. Two assessments per year were conducted, with a total of four assessments. DS was assessed based on the estimation of the percentage of the affected surface of the tree canopy using a 0–5 rating scale. Each scale value was referred as ‘category’ of severity (six categories in total) for further purposes of this study (*see* Section 4.3*. Sampling and fungal isolation*). The values of this scale have a linear relationship with the percentage of affected tissues (leaves and shoots) in order to satisfy the homogeneity of variances and normality for suitable statistical analysis [34]. The equivalences between the values of the scale and the percentage of affected surface of the tree canopy are: 0 = 0%, 1 = < 25%, 2 = 25–50%, 3 = 51–75%, 4 = 75–90%, 5 = ≥ 90%. The DS was assessed in June and September of each year (four evaluations in total), before, and after, harvest, respectively, and all the blocks of the whole of the experiment were evaluated each time. The relative area under the disease progress curve (RAUDPC) was calculated by the trapezoidal integration method from the disease severity values over time [35].

The dependent variables ‘final disease severity (%) and RAUDPC (%) were subjected to ANOVA to determine the differences in DS between irrigation treatments. Data were tested for normality and homogeneity of variances, and logarithmically transformed where necessary. Treatment means for the global analyses were compared using Fisher’s protected LSD test [36]. All the data were analysed using Statistix 10 [37].

### 4.3. Sampling and Fungal Isolation

Two almond trees per each category of disease severity (12 trees in total) were selected to temporarily monitor the fungi isolated from affected tissues. From each tree, branches and shoots showing dieback and cankers were collected in each disease assessment time. Samples were kept at 4 °C until being processed in the laboratory.

For fungal isolation, the outer bark of affected wood samples was removed, and were subsequently washed under running tap water. Little wood pieces were collected from the margin of the affected area of symptomatic samples or randomly selected across the wood section, in the case of asymptomatic samples (Category 0). All the wood pieces were surface disinfected by dipping into a 10% (vol/vol) solution of commercial bleach (Cl at 50 g l^−1^) for 2 min. Subsequently, they were air dried on sterile filter paper and plated onto malt extract agar (MEA) [20 g of MEA (Merck KGaA, Darrmstadt, Germany), 20 g of agar (Rokoagar AF LAB, ROKO Industries, Llanera, Asturias, Spain; 1 l of sterile distilled water (SDW)] supplemented with 0.5 g l^−1^ of streptomycin sulphate (Sigma-Aldrich, St. Louis, MO, USA) (MEAS). From each category of severity, a total of 168 wood pieces, obtained from the margin of the affected tissues, were plated on Petri dishes for fungal isolation [attempts of isolation (wood pieces) onto Petri dishes: 2 trees per disease category × 3 Petri dishes per tree × 7 wood pieces per Petri dish × 4 sampling moments]. Petri dishes were incubated for 5 to 14 days at 25 °C in darkness and they were examined daily removing the isolation attempts (wood pieces) contaminated by saprophytes (i.e., *Alternaria* spp. *Penicillium* spp., etc.) by cutting the agar up to 1-cm-radio beyond the colony margin using a sterile scalpel. It was necessary to prevent the contamination of the whole agar surface of the Petri dishes before our target fungi developed onto MEAS, due to the low mycelial growth rate of some expected fungi for isolation.

When the colonies were large enough to be examined, hyphal tips from the margin of the fungal colonies were transferred to the potato dextrose agar (PDA; Difco Laboratories^®^, Detroit) in order to obtain pure cultures. They were all incubated as previously described, and were grouped into four fungal groups (families) according to colony colour and mycelial growth development of each: Botryosphaeriaceae (light to dark grey, fast growing mycelium), *Collophorina* spp. (reddish to beige, very slow growing mycelia), *Cytospora* (beige to olive grey, middle-slow growing mycelium), and *Diaporthe* (beige-white, middle-fast growing mycelium). These preliminary morphological observations were helpful in selecting 30 representative isolates that were subsequently identified by molecular tools (Table 1). All the isolates were single-spored by a serial dilution method and they were registered and maintained at 4 °C in darkness (Fungal collection of the Department of Agronomy, University of Cordoba, Spain).

### 4.4. Assessment of Consistency and Frequency of Isolated Fungi

The consistency of isolation (%) of each isolate was calculated as the number of positive attempts of isolation (wood pieces) of a given fungus divided by the total attempts of isolation in the whole of the experiment [Consistency of isolation = (Nº. of positive wood pieces /168) × 100; where 168 is the total attempts of isolation (wood pieces) per category of disease severity in the whole of the experiment obtained as follow; 21 wood pieces per tree × 2 trees of each disease category × 2 years of evaluation × 2 sampling times per year]. The frequency of isolation (%) of each fungal species was estimated as the ratio between the number of trees from which each species was isolated and the total of sampled trees (12 trees). Additionally, the *in planta* abundance (biomass) of the studied fungal species and the severity of the dieback symptoms (category) was also compared by Pearson’s linear correlation (*n* = 5; data from Category 5 was excluded since only saprophytes were isolates) using Statistix 10 [37].

### 4.5. Molecular Identification of Isolated Fungi

#### 4.5.1. DNA Extraction

Mycelial tissues of the 30 isolated fungi (Table 1) previously grown on PDA were ground by means the FastPrep^®^-24 grinder machine (MP Biomedicals, Santa Ana, CA, USA). Subsequently, genomic DNA was extracted using the E.Z.N.A^®^ Fungal DNA Kit (OMEGA BioTek, Norcross, GA, USA). A MaestroNano^®^ spectrophotometer (MaestroGen, Taiwan) was used to determine the concentration and purity of the extracted DNA.

#### 4.5.2. PCR Analysis and Sequencing

The 5.8S nuclear ribosomal gene with two flanking internal transcribed spacers (ITS) was amplified for all the 30 isolated fungi. Subsequently, part of the beta-tubulin (TUB) gene, part of the translation elongation factor 1-alpha (EF) and/or a 200-bp intron of the glyceraldehyde-3-phosphate dehydrogenase (GAPDH) were amplified for the different isolates according to the necessities to complete the further phylogenetic analysis. To this end, the protocols described in the literature for each family and genus were followed to identify our fugal isolates (Table 1 and Table 3). The PCRs were performed in a total volume of 25 µl [20 ng of genomic DNA, 5 µl of 5× My Taq Reaction Buffer and 0.13 µl of My Taq DNA Polymerase (Bioline)]. Additionally, 0.4 or 0.2 μM (each) primer was added for the ITS; or for the TUB, EF, and GAPDH PCRs, respectively. A negative control was included in all PCRs using ultrapure water instead of DNA. Primer pairs and PCR cycling programs used to amplify each locus are shown in Table 3. Ultrapure water was used instead of DNA as negative control. A MyCycler™ Thermal Cycler (BIO-RAD) was used to conduct the PCRs.

Electrophoresis of the amplification products from PCR was conducted on a 1.5% (w/v) agarose gel stained with RedSafe^TM^ (Intron Biotechnology). A 100-bp DNA molecular weight marker (Ladder-GTP, gTPbio) was used, and the agarose gel was visualized under UV. Finally, the PCR products were purified by means the MEGAquick-spin^TM^ Total Fragment DNA Purification kit (INTRON Biotechnology). The resulting amplicons were sequenced in both directions [Central Service Support Research (SCAI) of the UCO (Spain)].

#### 4.5.3. Phylogenetic Analysis

Consensus sequences from DNA sequences generated with forward and reverse primers were obtained with the SeqMan software (DNASTART Lasergen SeqMan^®^ v. 7.0.0, Madison, WI, USA). They were compiled into a single FASTA file format. Subsequently, they were BLAST searched in GenBank (http://www.ncbi.nlm.nih.gov/genbank/) to determine the close related species for each sequence.

Firstly, a neighbor-joining (NJ) analysis was performed individually for each locus. It was useful to determine whether the sequence datasets were congruent and combinable (*data not shown*). To this end, the maximum composite likelihood method with 2000 bootstrap replications was used. Genetic distances were calculated using the Kimura 2-parameter mode and tree topologies of 70% reciprocal bootstrap generated individually for each locus were compared visually. The data of different loci were combined into single concatenated datasets when no supported nodes were in conflict.

Independent phylogenetic analyses were conducted for the isolates of each fungal group (family), previously established according to their main morphological characteristics and Blast analysis. The combined alignment of the ITS and TUB loci was analysed in order to infer the phylogeny of isolates belonging to Botryosphaeriaceae (*Dataset I-A*). Additionally, a little phylogeny combining ITS and EF loci was also conducted into Botryosphaeriaceae group to confirm the identification of *Neoscytalidium* sp. Isolate (*Dataset I-B*). In the case of Diaporthaceae, the combined alignment of the EF, TUB and ITS loci was conducted (*Dataset II*). Isolates belonging to Tympanidaceae were identified by means the combined alignment of the ITS, EF and GAPDH loci (*Dataset III*). Finally, the combined alignment of the ITS and EF loci was performed to infer the phylogeny of the isolates belonging to Valsaceae (*Dataset IV*). For each multilocus alignment, data of the reference taxa (including outgroup) downloaded from GenBank and the number of the taxa included in this study are shown in Table 1, and Table 2, respectively.

The reference Genbank taxa were selected based on their high similarity with our query sequences using MegaBLAST [38] and they were added and aligned with our sequences by Clustal W. Maximum parsimony (MP) analyses were conducted using MEGA version 7.0 software [38], and they were performed by means the Tree-Bisection-Regrafting (TBR) algorithm with search level one. The initial trees were obtained by the random addition of sequences (10 replicates). The gaps and missing data were treated as complete deletions. A total of 1,000 bootstrap replications were done to ensure the robustness of the topology [39]. Tree length (TL), consistency index (CI), retention index (RI), homoplasy index (HI) and rescaled consistency index (RC) were calculated for each resulting MP tree.

Additionally, Markov chain Monte Carlo (MCMC) methods were used to perform Bayesian inference (BI) analyses by means the software MrBayes v.3.2.6 [40]. They were useful for estimating the posterior probability of trees. The best fit models of the evolution used for each gene partition were also determined by MEGA v. 7.0 [38]. Two analyses with four MCMC chains each were run simultaneously for 1 × 10^7^ generations, starting from a random tree topology. The trees were sampled every 100 generations, and the “temperature” parameter was set to 0.2. The first 25% of the saved trees was discarded as the burn-in phase of the analysis. The sequences derived in this study were uploaded at GenBank (Table 1).

**Table 3:** Primer pairs and PCR conditions used for the amplification of the genes included in this study.

Gene ^1^	Primer Pairs	PCR Cycling Program (Tª-Time)						References
		Initial Denaturation	Amplification				Final extension	
			N° of Cycles	Denaturation	Annealing	Extension		
ITS	ITS4/ITS5	95 °C-3 min	35	95 °C-30 s	48 °C-30 s	72 °C-45 s	72 °C-10 min	[41]
TUB	Bt2a/Bt2b	95 °C-3 min	35	95 °C-15 s	55 °C-15 s	72 °C-45 s	72 °C-7 min	[42]
EF	EF1-728F/EF1-986R	95 °C-3 min	35	95 °C-30 s	50 °C-30 s	72 °C-45 s	72 °C-10 min	[43]
GAPDH	GDF1/GDR1	94 °C-5 min	40	95 °C-15 s	52 °C-15 s	72 °C-10 s	72 °C-7 min	[44]

^1^ ITS = internal transcribed spacer; TUB = β-tubulin; EF = translation elongation factor 1-α; GAPDH = a 200-bp intron of the glyceraldehyde-3-phosphate dehydrogenase.

## Figures and Tables

**Table 1:** Fungal isolates used in the phylogenetic analysis and their corresponding GenBank accession numbers.

Species	Isolate ^1^	Consistency of isolation (%) ^2^	Host/Cultivar	Collector	Date ^3^	GenBank Accession no.^4^			
						ITS	TUB	EF	GAPDH
Botryosphaeriaceae analyses									
*Botryosphaeria dothidea*	ColPat-607	3.6	*Prunus dulcis* cv. Lauranne	C. Agustí-Brisach & A. Trapero	06/12/2018	MT303980	MT309728	-	-
	ColPat-610	22.6	*Prunus dulcis* cv. Lauranne	C. Agustí-Brisach & A. Trapero	06/12/2018	MT303982	MT309730	-	-
	ColPat-652	7.2	*Prunus dulcis* cv. Lauranne	C. Agustí-Brisach & A. Trapero	09/13/2018	MT303983	MT309731	-	-
	ColPat-653	6.4	*Prunus dulcis* cv. Lauranne	C. Agustí-Brisach & A. Trapero	09/13/2018	MT303984	MT309732	-	-
	ColPat-654	8.2	*Prunus dulcis* cv. Lauranne	C. Agustí-Brisach & A. Trapero	09/13/2018	MT303985	MT309733	-	-
	ColPat-657	4.8	*Prunus dulcis* cv. Lauranne	C. Agustí-Brisach & A. Trapero	09/13/2018	MT303986	MT309734	-	-
	ColPat-658	6.4	*Prunus dulcis* cv. Lauranne	C. Agustí-Brisach & A. Trapero	09/13/2018	MT303987	MT309735	-	-
	ColPat-764	11.9	*Prunus dulcis* cv. Lauranne	C. Agustí-Brisach & A. Trapero	06/13/2019	MT303988	MT309736	-	-
	ColPat-765	9.1	*Prunus dulcis* cv. Lauranne	C. Agustí-Brisach & A. Trapero	06/13/2019	MT303989	MT309737	-	-
	ColPat-794	16.0	*Prunus dulcis* cv. Lauranne	C. Agustí-Brisach & A. Trapero	09/11/2019	MT303991	MT309739	-	-
	ColPat-795	23.8	*Prunus dulcis* cv. Lauranne	C. Agustí-Brisach & A. Trapero	09/11/2019	MT303992	MT309740	-	-
	ColPat-796	19.1	*Prunus dulcis* cv. Lauranne	C. Agustí-Brisach & A. Trapero	09/11/2019	MT303993	MT309741	-	-
	ColPat-797	17.9	*Prunus dulcis* cv. Lauranne	C. Agustí-Brisach & A. Trapero	09/11/2019	MT303994	MT309742	-	-
	**CBS 100564, PD 97,14304**	-	*Paeonia* sp	P. Vink	nd	KX464085	KX464781	-	-
	**PD4, 3626**	-	*Prunus dulcis*	T.J. Michailides	8/2005	GU251091	GU251751	-	-
	**PD33,3657**	-	*Prunus dulcis*	T.J. Michailides	8/2005	GU251093	GU251753	-	-
	**PD68,3623**	-	*Prunus dulcis*	T.J. Michailides	8/2005	GU251095	GU251755	-	-
	**PD107, 809**	-	*Prunus dulcis*	T.J. Michailides	8/2005	GU251097	GU251757	-	-
	**PD122, A2.1**	-	*Prunus dulcis*	T.J. Michailides	5/2007	GU251098	GU251758	-	-
	**PD146, A27**	-	*Prunus dulcis*	T.J. Michailides	5/2007	GU251099	GU251759	-	-
*Diplodia juglandis*	**CBS 188.87**	-	*Juglans regia*	nd	nd	EU673316	EU673119	-	-
*Diplodia mutila*	**6B99**	-	*Juglans regia*	nd	5/31/2011	KF778791	KF778886	-	-
	**CBS 112553^T^**	-	*Vitis vinifera*	A.J.L. Phillips	nd	AY259093	DQ458850	-	-
*Diplodia seriata*	**CBS 112555^T^**	-	*Vitis vinifera*	A. J. L. Phillips	nd	AY259094	DQ458856	-	-
	**3H18**	-	*Juglans regia*	nd	nd	KF778796	KF778891	-	-
	**PD34, 3381**	-	*Prunus dulcis*	T.J. Michailides	7/2004	GU251111	GU251771	-	-
	**PD50, 3348**	-	*Prunus dulcis*	T.J. Michailides	8/2004	GU251113	GU251773	-	-
	**Mz-F1**	-	*Malus domestica*	nd	nd	KU942427	KU976444	-	-
*Dothiorella iberica*	**CBS 115041^T^**	-	*Quercus ilex*	J. Luque	12/2009	AY573202	EU673096	-	-
	**5G97**	-	*Juglans regia*		12/13/2010	KF778808	KF778903	-	-
	**UCD1448SLO**	-	nd	nd	nd	EF202009	EF202016	-	-
	**UCRDI3**	-	*Prunus dulcis*	nd	nd	KP012591	KP067201	-	-
*Dothiorella sarmentorum*	**CBS 164.33**	-	nd	nd	nd	KX464127	KX464881	-	-
	**PD78, 3797**	-	*Prunus dulcis*	T.J. Michailides	8/2006	GU251169	GU251829	-	-
	**PD79, 3795**	-	*Prunus dulcis*	T.J. Michailides	8/2006	GU251170	GU251830	-	-
*Lasiodiplodia citricola*	**6I34**	-	*Juglans regia*	nd	10/6/2011	KF778809	KF778904	-	-
*Lasiodiplodia theobromae*	**CBS 124.13**	-	nd	J.J. Taubenhaus	nd	DQ458890	DQ458858	-	-
*Macrophomina phaseolina*	**PD112, A28.1**	-	*Prunus dulcis*	T.J. Michailides	5/2007	GU251105	GU251765	-	-
*Neofusicoccum australe*	**Y264-21-1**	-	*Vitis vinifera*	nd	nd	JF437920	JF437922	-	-
*Neofusicoccum mediterraneum*	ColPat-605	7.2	*Prunus dulcis* cv. Lauranne	C. Agustí-Brisach & A. Trapero	06/12/2018	MT303979	MT309727	-	-
	ColPat-799	4.8	*Prunus dulcis* cv. Lauranne	C. Agustí-Brisach & A. Trapero	09/11/2019	MT303995	MT309743	-	-
	**CBS 121718^T^; PD312**	-	*Eucalyptus* sp.	nd	6/2006	GU251176	GU251836	-	-
	**PD48, 3483**	-	*Prunus dulcis*	T.J. Michailides	9/2004	GU251186	GU251846	-	-
	**PD49, 3227**	-	*Prunus dulcis*	T.J. Michailides	6/2004	GU251187	GU251847	-	-
	**PD55, 2953**	-	*Prunus dulcis*	T.J. Michailides	1/2004	GU251189	GU251849	-	-
	**Bot-04**	-	*Vitis vinifera* cv. Pedro Ximénez	C. Agustí- Brisach and A. Trapero	2016	MG745841	MG745803	-	-
	**1H96**	-	*Juglans* *regia*	nd	9/15/2006	KF778811	KF778906	-	-
	**CAA 002**	-	*Pistacia vera* cv. Kerman	T.J. Michailides	nd	EU017537	KX505925	-	-
	**Y546-2-1**	-	*Vitis vinifera*	nd	nd	JF437919	JF437921	-	-
*Neofusicoccum nonquaesitum*	**PD86, A9**	-	*Prunus dulcis*	T.J. Michailides	5/2007	GU251156	GU251816	-	-
	**PD90, A42**	-	*Prunus dulcis*	T.J. Michailides	5/2007	GU251157	GU251817	-	-
*Neofusicoccum parvum*	ColPat-608	14.3	*Prunus dulcis* cv. Lauranne	C. Agustí-Brisach & A. Trapero	06/12/2018	MT303981	MT309729	-	-
	**CMW9081^T^**	-	*Pinus nigra*	G. J. Samuels	nd	AY236943	AY236917	-	-
	**1L83**	-	*Juglans* *regia*	nd	11/4/2005	KF778854	KF778949	-	-
	**PD17, 3621**	-	*Prunus dulcis*	T.J. Michailides	8/2005	GU251143	GU251803	-	-
	**PD39, 3656**	-	*Prunus dulcis*	T.J. Michailides	8/2005	GU251144	GU251804	-	-
*Neoscytalidium dimidiatum*	ColPat-792	25.0	*Prunus dulcis* cv. Lauranne	C. Agustí-Brisach & A. Trapero	09/11/2019	MT303990	MT309738	-	-
	**Kale4-C**	-	*Prunus armeniaca*	E. Oksal	2018	MK788362	MK803352	-	-
	**Arp2-D**	-	*Vitis vinifera*	E. Oksal	2018	MK813852	MK816354	-	-
	**2-D61**	-	*Ficus carica*	M. Nouri	2016	MG021572	MG021515	-	-
	**CBS 145.78**	-	nd	nd	nd	MH861121	KF531796	-	-
	**COUFAL0146**	-	*Nopalea rochenillifera*	nd	nd	MH251955	MH251971	-	-
*N. novaehollandiae*	**CBS122071**	-	*Crotalaria medicaginea*	nd	nd	EF585540	-	EF585580	-
	**CBS122610**	-	*Acacia synchronicia*	nd	nd	EF585536	-	EF585578	-
*Diaporthe asheicola*	**CBS 136967**	-	*Vaccinium ashei*	nd	nd	KJ160562	KJ160518	-	-
**Diaporthaceae analyses**									
*Diaporthe acaciigena*	**CBS 129521^T^**	-	*Acacia retinodes*	P.W. Crous, I.G. Pascoe & J. Edwards	nd	KC343005	KC343973	KC343731	-
*Diaporthe alleghaniensis*	**CBS 495.72^T^**	-	*Betuta alleghaniensis*	nd	nd	FJ889444	KC343975	GQ250298	-
*Diaporthe alnea*	**CBS 146.46^T^**	-	*Alnus* sp.	S. Truter	nd	KC343008	KC343976	KC343734	-
*Diaporthe ambigua*	**CBS 114015**	-	nd	nd	nd	MH862953	KC343978	KC343736	
*Diaporthe arctii*	**DP0482**	-	*Arctium lappa*	W. Jaklitsch	nd	KJ590736	KJ610891	KJ590776	-
*Diaporthe australafricana*	**CBS 111886^T^**	-	*Vitis vinifera*	L. Mostert	nd	KC343038	KC344006	KC343764	-
*Diaporthe chamaeropis*	**CBS 753.70**	-	*Spartium junceum*	J.A. von Arx	nd	KC343049	KC344017	KC343775	-
*Diaporthe cinerascens*	**CBS 719.96**	-	*Ficus carica*	E. Ilieva	nd	KC343050	KC344018	KC343776	-
*Diaporthe cuppatea*	**CBS 117499**	-	*Aspalathus linearis*	J.C. Janse van Rensburg	nd	MH863021	KC344025	KC343783	-
*Diaporthe cynaroidis*	**CBS 122676^T^**	-	*Protea cynaroides*	S. Marincowitz	nd	NR111846	KC344026	KC343784	-
*Diaporthe eres*	**CBS 287.74**	-	*Sorbus aucuparia*	W.M. Loerakker	nd	KC343084	KC344052	KC343810	-
*Diaporthe hickoriae*	**CBS 145.26^T^**	-	*Carya glabra*	L.E. Wehmeyer	nd	NR103699	KC344086	GQ250309	-
*Diaporthe inconspicua*	**CBS 133813^T^**	-	*Maytenus ilicifolia*	R.R. Gomes	nd	NR111849	KC344091	KC343849	-
*Diaporthe infecunda*	**CBS 133812^T^**	-	*Schinus terebinthifolius*	J. Lima	nd	NR111850	KC344094	KC343852	-
*Diaporthe lusitanicae*	**CBS 123212**	-	nd	nd	nd	MH863279	KC344104	KC343862	-
*Diaporthe neotheicola*	ColPat-762	21.4	*Prunus dulcis* cv. Lauranne	C. Agustí-Brisach & A. Trapero	06/13/2019	MT304007	MT309745	MT309762	-
	ColPat-763	17.1	*Prunus dulcis* cv. Lauranne	C. Agustí-Brisach & A. Trapero	06/13/2019	MT304008	MT309746	MT309763	-
	**CBS 111553^T^**	-	*F. vulgare*	Alan Phillips	nd	NR145303	KC344069	KC343827	-
	**6I30**	-	*Juglans* *regia*	T.J. Michailides	10/6/2011	KF778871	KF778966	KF779061	-
	**CAA816**	-	*Vaccinium corymbosum*	nd	nd	MK792314	MK837934	MK828083	
	ColPat-445	-	*Juglans regia* cv. Tulare	C. Agustí-Brisach & A. Trapero	07/14/2017	MK522106	MK447993	MK490932	
*Diaporthe novem*	**CBS 127270^T^**	-	*Glycine max*	T. Duvnjak	nd	NR111855	KC344124	KC343882	
*Diaporthe phaseolorum*	**AR4203**	-	*Phaseolus vulgaris*			KJ590738	KJ610893	KJ590739	-
*Diaporthe rhusicola*	ColPat-606	25.0	*Prunus dulcis* cv. Lauranne	C. Agustí-Brisach & A. Trapero	06/12/2018	MT304006	MT309744	MT309761	-
	**6I14**	-	*Prunus dulcis*	T.J. Michailides	9/12/2011	KF778872	KF778967	KF779062	
	**6I15**	-	*Prunus dulcis*	T.J. Michailides	9/12/2011	KF778873	KF778968	KF779063	-
	**6I31**	-	*Juglans regia*	T.J. Michailides	10/06/2011	KF778874	KF778969	KF779064	-
	**6I43**	-	*Juglans regia*	T.J. Michailides	10/06/2011	KF778875	KF778970	KF779065	-
*Diaporthe vaccinii*	**CBS 160.32^T^**	-	*Oxycoccus macrocarpos*	C.L. Shear	nd	NR103701	KC344196	KC343954	
*Diaporthella corylina*	**CBS 121124**	-	*Corylus* sp.	nd	nd	KC343004	KC343972	KC343730	-
*Phomopsis amygdali*	**CBS 126679^T^**	-	*Prunus dulcis*	nd	nd	KC343022	KC343990	KC343748	-
	**CBS 115620**	-	*Prunus persica*	nd	nd	KC343020	KC343988	KC343746	-
**Tympanidaceae analyses**									
*Collophorina africana*	**CBS 120872^T^**		*Prunus salicina*	U. Damm	nd	GQ154570	-	GQ154643	GQ154648
	**GLMC 1736**		*Prunus domestica*	nd	nd	MK314542	-	MK314507	MK314474
*Collophorina badensis*	**GLMC 1684^T^**		*Prunus domesica*	nd	nd	MK314546	-	MK314503	MK314482
*Collophorina capensis*	**CBS 120879**		*Prunus salicina*	U. Damm	nd	GQ154571	-	GQ154644	GQ154649
*Collophorina germanica*	**GLMC 1445^T^**		*Prunus avium*	nd	nd	MK314550	-	MK314515	MK314477
*Collophorina hispanica*	ColPat-651	2.4	*Prunus dulcis* cv. Lauranne	C. Agustí-Brisach & A. Trapero	09/13/2018	MT303996	-	MT309747	MT309754
	ColPat-655	9.5	*Prunus dulcis* cv. Lauranne	C. Agustí-Brisach & A. Trapero	09/13/2018	MT303997	-	MT309748	MT309755
	ColPat-759	9.5	*Prunus dulcis* cv. Lauranne	C. Agustí-Brisach & A. Trapero	06/13/2019	MT303998	-	MT309749	MT309756
	ColPat-760	9.5	*Prunus dulcis* cv. Lauranne	C. Agustí-Brisach & A. Trapero	06/13/2019	MT303999	-	MT309750	MT309757
	ColPat-761	8.3	*Prunus dulcis* cv. Lauranne	C. Agustí-Brisach & A. Trapero	06/13/2019	MT304000	-	MT309751	MT309758
	ColPat-800	2.4	*Prunus dulcis* cv. Lauranne	C. Agustí-Brisach & A. Trapero	09/11/2019	MT304001	-	MT309752	MT309759
	ColPat-801	3.6	*Prunus dulcis* cv. Lauranne	C. Agustí-Brisach & A. Trapero	09/11/2019	MT304002	-	MT309753	MT309760
	**CBS 128566**	-	*Prunus dulcis*	J. Armengol	2010	JN808839	-	JN808850	JN808843
	**CBS 128568^T^**	-	*Prunus dulcis*	J. Armengol	2010	JN808841	-	JN808852	JN808845
	**CBS 128569**	-	*Prunus dulcis*	J. Armengol	2010	MH864962	-	JN808853	JN808846
*Collophorina neorubra*	**GLMC 929^T^**	-	*Prunus avium*	nd	nd	MK314533	-	MK314511	MK314485
*Collophorina paarla*	**CBS 120877**	-	*Prunus salicina*	U. Damm	nd	GQ154586	-	GQ154645	GQ154651
*Collophorina rubra*	**CBS 120873^T^, STE-U 6109**	-	*Prunus persica*	U. Damm	nd	NR119747		JN808855	JN808848
*Cadophora luteo-olivacea*	**CBS 141.41^T^**	-	*Prunus dulcis*	nd	nd	AY249066	-	KM497089	JN808849
**Valsaceae analyses**									
*Cytospora acaciae*	**CBS 468.69**	-	*Ceratonia siliqua*	nd	nd	MH859354	-	KX965181	-
*Cytospora amygdali*	**LH356**	-	*Prunus dulcis*	nd	nd	MG971852	-	MG971658	-
	**LH357^T^, CBS 144233**	-	*Prunus dulcis*	nd	nd	MG971853	-	MG971659	-
*Cytospora austromontana*	**CBS 116821**	-	*Eucalyptus pauciflora*	nd	nd	KY051796	-	KX965068	-
*Cytospora cabornacea*	**CBS 219.54**	-	*Ulmus* sp.	nd	nd	DQ243805	-	KX965164	-
*Cytospora californica*	**KARE1105**	-	*Prunus dulcis*	nd	nd	MG971947	-	MG971663	-
*Cytospora cedri*	ColPat-604	10.8	*Prunus dulcis* cv. Lauranne	C. Agustí-Brisach & A. Trapero	06/12/2018	MT304003	-	MT311983	-
	**CBS 196.97**	-	nd	nd	nd	KY051906	-	KX965154	-
*Cytospora ceratosperma*	**CBS 512.76**	-	*Fagus sylvatica*	nd	nd	KY051941	-	KX965184	-
*Cytospora chrysosperma*	**9E-33, CBS 144242**	-	*Camellia* sp.	nd	nd	MG971892	-	MG971602	-
*Cytospora eucalypti*	**KARE1585, CBS 144241**	-	*Prunus dulcis*	nd	nd	MG971907	-	MG971617	-
*Cytospora granati*	**6F-45^T^, CBS 144237**	-	*Punica granatum*	nd	nd	MG971799	-	MG971514	-
*Cytospora joaquinensis*	**KARE975^T^, CBS 144235**	-	*Populus deltoides*	nd	nd	MG971895	-	MG971605	-
*Cytospora longispora*	**10F-57^T^, CBS 144236**	-	*Prunus domestica*	nd	nd	MG971905	-	MG971615	-
*Cytospora mali*	**CBS 109499**	-	*Malus* sp.	nd	nd	KY051769	-	KX965048	-
*Cytosppora oleicola*	**KARE1021^T^, CBS 144248**	-	*Olea europaea*	nd	nd	MG971944	-	MG971660	-
*Cytospora parakantschavelii*	**KARE974, CBS 144243**	-	*Populus deltoides*	nd	nd	MG971898	-	MG971608	-
*Cytospora parapistaciae*	**KARE232**	-	*Pistacia vera*	nd	nd	MG971807	-	MG971522	-
	**KARE270^T^, CBS 144506**	-	*Pistacia vera*	nd	nd	MG971804	-	MG971519	-
*Cytospora parasitica*	**CFCC 53173**	-	*Berberis* sp.	nd	nd	MK673070	-	MK672957	-
*Cytospora pavettae*	**CBS 145562**	-	*Pavetta revoluta*	M.J. Wingfield	nd	MK876386	-	MK876497	-
*Cytospora pistaciae*	**KARE443^T^, CBS 144238**	-	*Pistacia vera*	nd	nd	MG971802	-	MG971517	-
*Cytospora plurivora*	**KARE1452^T^, CBS 144239**	-	*Olea europaea*	nd	nd	MG971861	-	MG971572	-
*Cytospora populicola*	**KARE973^T^, CBS 144240**	-	*Populus deltoides*	nd	nd	MG971891	-	MG971601	-
*Cytospora punicae*	**7C-09**	-	*Punica granatum*	nd	nd	MG971939	-	MG971650	-
*Cytospora rhodophila*	**CBS 190.42**	-	*Syringa* sp.	nd	nd	KY051901	-	KX965147	-
*Cytospora ribis*	**CBS 187.36**	-	*Ribes rubrum*	nd	nd	DQ243810	-	KX965144	-
	**CFCC 50039**	-	*Platycladus orientalis*	Xinlei Fan	nd	KR045642	-	KU710931	-
*Cytospora sacculus*	**CFCC 89626**	-	*Juglans regia*	Xinlei Fan	nd	KR045647	-	KU710934	-
	**CBS 116855**	-	*Quercus alba*	nd	nd	KY051824	-	KX965091	-
*Cytospora sorbicola*	**4L-58**	-	*Prunus domestica*	nd	nd	MG971839	-	MG971553	-
*Cytospora* sp. 1	ColPat-609	14.3	*Prunus dulcis* cv. Lauranne	C. Agustí-Brisach & A. Trapero	06/12/2018	MT304004	-	MT311984	-
*Cytospora* sp. 2	ColPat-656	2.0	*Prunus dulcis* cv. Lauranne	C. Agustí-Brisach & A. Trapero	09/13/2018	MT304005	-	MT311985	-
*Diaporthella corylina*	**CBS 121124**	-	*Corylus* sp.	L.N. Vassiljeva	nd	KC343004	-	KC343730	-
*Valsa germanica*	**CBS 195.42**	-	nd	nd	nd	KY051902	-	KX965151	-

^1^ Sequences from GenBank used in the phylogenetic analysis indicated in bold type. T = Ex-type isolates; AR, DP: Isolates in culture collection of Systematic Mycology and Microbiology Laboratory, USDA-ARS, Beltsville, Maryland, USA; CAA = A. Alves, Universidade de Aveiro, Portugal; CBS: Culture collection of the Centraalbureau voor Schimmelcultures, Fungal Biodiversity Centre, Utrecht, The Netherlands; CFCC = China Forestry Culture Collection Center; CMW = Culture collection of the Forestry and Agricultural Biotechnology Institute (FABI), University of Pretoria, Pretoria, South Africa; ColPat = ‘Colección Patología’, Department of Agronomy, University of Cordoba, Spain; KARE= Collections of the Department of Plant Pathology at the Kearney Agricultural Research and Extension Centre of the University of California, Parlier, CA.; PD: Plant Protection Service, Wageningen, The Netherlands; STE-U = University of Stellenbosch, South Africa. ^2^ The consistency of isolation (%) of each isolate was calculated as the number of positive attempts of isolation (wood pieces) of a given fungus divided by the total attempts of isolation in the whole of the experiment [Consistency of isolation = (Nº. of positive wood pieces /168) × 100; where 168 is the total attempts of isolation (wood pieces) per category of disease severity in the whole of the experiment obtained as follow: 21 wood pieces per tree × 2 trees of each disease category × 2 years of evaluation × 2 sampling times per year]. ^3^ Collection date: month/day/year; n/d: non-determined. ^4^ ITS = internal transcribed spacer, TUB = β-tubulin-2 gene regions, EF = translation elongation 1-α, GAPDH = 200-bp intron of the glyceraldehyde-3-phosphate dehydrogenase.

**Table 2:** Number of taxa, genes and statistical information of the different analyses performed in this study: Bayesian (IB) and Maximum Parsimony Analyses (MP).

Dataset/Phylogenetic Analyses	Number of Taxa			Gene			Bayesian Analyses		Maximum Parsimony Analyses										
									Characters ^2^					MP Tree ^3^					
	In Study	GenBank (incl.outgroups)		Combination	Boundaries		Best Fit Model ^1^		T	PI	PNI	C		Nº.Trees	TL	CI	RI	HI	RC
I-A/Botryosphaeriaceae	17	43		ITS/ TUB	1-503/ 504-921		K2+G/ T92+G		733	119	133	481		4	375	0.677	0.940	0.323	0.636
I_B/*Neoscytalidium dimidiatum*	1	8		ITS/ EF	1-490/ 491-711		T92/ K2		684	9	244	431		9	258	0.857	0.833	0.143	0.714
II/Diaporthaceae	3	30		EF/TUB/ ITS	1-254/ 255-739/ 740-1227		K2+G/ T92+I/ K2+G+I		993	301	124	568		1	1046	0.551	0.769	0.449	0.424
III/Tympanidaceae	7	12		ITS/ EF/ GAPDH	1-482/ 483-667/ 668-805		K2+G/ K2+I/ K2+I		719	100	143	476		10	334	0.850	0.919	0.150	0.782
IV/Valsaceae	3	32		ITS/ EF/	1-557/ 558-821		K2+G+I/ HK4+G		613	171	73	369		1	743	0.469	0.742	0.531	0.348

^1^ Best fit nucleotide substitution models determined by MEGA v. 7.0, used for each gene partition to perform Bayesian Inference analyses using MrBayes v.3.2.6. ^2^ Numbers of total characters (positions) in the final dataset (T), parsimony-informative characters (PI), parsimony-uninformative characters (PNI) and conserved sites (C), processed in each analysis; all positions containing gaps and missing data were eliminated. ^3^ Total of equally most parsimonious trees obtained for each MP analyses: Nº of Tree, tree length (TL), consistency index (CI), retention index (RI), homoplasy index (HI) and rescaled consistency index (RC).
